# A Novel Group Decision-Making Method Based on Sensor Data and Fuzzy Information

**DOI:** 10.3390/s16111799

**Published:** 2016-10-28

**Authors:** Yu-Ting Bai, Bai-Hai Zhang, Xiao-Yi Wang, Xue-Bo Jin, Ji-Ping Xu, Ting-Li Su, Zhao-Yang Wang

**Affiliations:** 1 School of Automation, Beijing Institute of Technology, Beijing 100081, China; byting@bit.edu.cn (Y.-T.B.); wangzhaoyang@bit.edu.cn (Z.-Y.W.); 2 School of Computer and Information Engineering, Beijing Technology and Business University, Beijing 100048, China; wangxy@btbu.edu.cn (X.-Y.W.); jinxuebo@th.btbu.edu.cn (X.-B.J.); xujp@th.btbu.edu.cn (J.-P.X.); sutingli@btbu.edu.cn (T.-L.S.)

**Keywords:** group decision making, Vague set, water environment management, algal bloom remediation

## Abstract

Algal bloom is a typical phenomenon of the eutrophication of rivers and lakes and makes the water dirty and smelly. It is a serious threat to water security and public health. Most scholars studying solutions for this pollution have studied the principles of remediation approaches, but few have studied the decision-making and selection of the approaches. Existing research uses simplex decision-making information which is highly subjective and uses little of the data from water quality sensors. To utilize these data and solve the rational decision-making problem, a novel group decision-making method is proposed using the sensor data with fuzzy evaluation information. Firstly, the optimal similarity aggregation model of group opinions is built based on the modified similarity measurement of Vague values. Secondly, the approaches’ ability to improve the water quality indexes is expressed using Vague evaluation methods. Thirdly, the water quality sensor data are analyzed to match the features of the alternative approaches with grey relational degrees. This allows the best remediation approach to be selected to meet the current water status. Finally, the selection model is applied to the remediation of algal bloom in lakes. The results show this method’s rationality and feasibility when using different data from different sources.

## 1. Introduction

Decision-making is the core of management and has been widely applied in the economy, industry and engineering [1,2,3,4]. It usually depends on administrators’ knowledge and experience but increasingly relies on objective data and information. Various types of water quality sensors can provide real-time monitoring data on the management of rivers and lakes. Previous management only focused on intuitional information from the monitoring data, such as the water quality indexes’ normal conditions and those that were out of the limits, which lacks deep and secondary analysis [5]. Administrators have been concerned about how to obtain implicit information from the sensor data for effective management and decision-making.

In a broad sense, decision-making includes the process of presenting questions, setting goals, and designing and selecting approaches, while in the narrow sense, it means selecting the best approach among several alternatives [6]. This is usually achieved by the classic multi-attribute decision-making method. It first establishes a set of evaluation attributes and then sorts the different alternative options using provided evaluation values. The multi-attribute method can solve the irrational analysis problem and improve objectivity to a certain extent. However, only a part of the evaluated attribute values that have direct connection with the final decision is from objective data in most cases, and most values are given by human interpretation. The knowledge and experience of the administrators significantly impact the decision results. Therefore, minimizing the human effect and enhancing the role of data becomes the key to increasing the rational degree of decision-making.

Globally, nearly 40% of lakes and rivers suffer from different levels of water eutrophication. Eutrophic lakes and rivers account for 24.6% of the major lakes in China [7]. Algal bloom is the typical result of water eutrophication and is caused by the great increase of nitrogen, phosphorus and other nutrients when lakes and rivers are polluted [8,9]. Both Lake Constance and Lake Erie have algal bloom pollution, and in China, many lakes and reservoirs in the middle and lower parts of both the Yangtze River and the Yellow River also break out in algal blooms very frequently, and the problem is particularly serious in Lake Tai, Chao Lake and Dian Lake. Algal bloom has become a worldwide public hazard, seriously threatening local water safety and environmental maintenance.

Various remediation approaches have been studied to reduce the harm of algal bloom, including physical, chemical and biological methods. These methods focus on specific remediation principles instead of selection on the basis of different environments, meaning that administrators must select remediation approaches subjectively and in light of personal experience. It is difficult for such selection to take all influential factors into account, and thus the decision may be one-sided, inefficient and a waste of resources. It has been problematic to scientifically select remediation approaches according to the status of the water environment.

Decision-making theories and methods have been applied in various fields, but research on the remediation of algal bloom is still in the initial stage. Existing decision-making methods on remediation are essentially multi-attribute methods. The literature [10,11] has analyzed attributes such as risk value and obtained the decision results with Bayesian and fuzzy Bayesian theory under constraint conditions. Other literature [12,13,14] has divided the attributes into different layers consisting of objectives and parameters. Optimal weights and attribute value synthesis were obtained with information entropy and grey theory. There are two main disadvantages in this research: First, decision-making opinions are only given by one expert, which tends to result in a constrained series of options, considering the limits of one person’s knowledge. Second, the attributes are all subjectively qualitative information as delivered by experts, expressed as the effects of the approaches to each attribute. The existing decision-making process fully depends on subjective information which can only reflect the general features of the approaches and lacks relevance to the real-time environmental status.

For the problems above, more experts should be invited to provide decision opinions so that the basis of the decision can be more comprehensive by aggregating the opinions. Moreover, monitoring data from the water quality sensors should be used to include the real-time status as an important basis for the selection of the remediation approach.

A novel solution is proposed to solve the problems of simplex information and the lack of alignment with real-time status. The main method fused the sensor data with the fuzzy evaluation information expressed by the Vague set. The Vague set [15] is a powerful tool to accommodate linguistic and grade variables by providing supportive, opposed and uncertain information. In this paper, the similarity measurement of the Vague value is modified as the basis of the novel aggregation method of group opinions. Experts’ opinions are aggregated to indicate the ability of the alternatives to improve different water quality parameters. Then, the approach for the current status is selected by correlating the monitoring data with the fuzzy evaluation information. The presented group decision-making method in the remediation approach selection makes full use of monitoring data and evaluation opinions and effectively combines the qualitative and quantitative information with the novel Vague set computation method, which helps increase the effectiveness of the decision result.

## 2. Group Decision-Making Method

### 2.1. General Solution of Group Decision-Making

**Figure 1:**
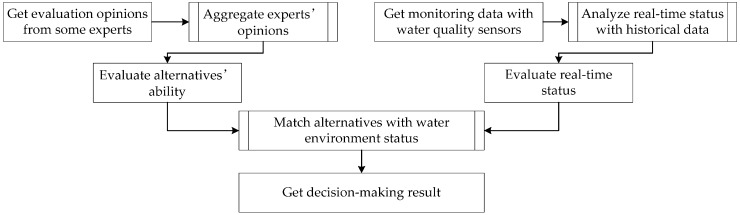
The process of the group decision-making method.

Decision-making for the remediation of algal bloom means selecting the best approach from the alternatives according to the current water environment when or before the algal bloom breaks out. The group decision-making method in this paper increases decision sources by inviting more experts. The sensors’ monitoring data are fused with the fuzzy evaluation information. With this in mind, the general solution of the group decision-making method is shown in Figure 1.

The experts evaluate the alternatives from each attribute, and the opinions are aggregated using the method in this paper. The evaluation of the approaches can reflect the alternatives’ ability to improve different attributes.

The current status of the rivers and lakes is analyzed using both the water quality sensors’ monitoring data and historical data. The alternatives are matched with the current status, forming the basis from which the best approach to the current environment is selected.

The main points of the decision-making method include the aggregation of group opinions, the evaluation of approaches and the matching of the approaches to the real-time status. The concrete methods for performing each of these steps are introduced in Section 2.2, Section 2.3 and Section 2.4.

### 2.2. Optimal Similarity Aggregation Model of Group Opinions

The purpose of aggregating the opinions of the expert group is to obtain the final opinion closest to all opinions so that all experts can be considered. This paper proposes a novel optimal similarity aggregation model of group opinions in the Vague set. It aims to minimize the sum of the inconsistent degree between the aggregated opinion and the individual opinions. Assume *K* is the number of individuals, *R_k_* is the *k*-th individual’s evaluation opinion expressed in the Vague set, and *R* is the aggregated opinion. *d_k_* is the individual’s relative consistent degree, which is an element of set *D*.

The similarity between *R_k_* and *R* is expressed as $M(R_{k},R)$. Then, the diversity degree can be defined as $c-M(R_{k},R)$, in which *c* is constant and c≥1. A model of the minimization problem is built to obtain the optimal aggregated opinion:

(1) $$\begin{array}{c} \min Q_{q,c}(D,R)=\sum_{k=1}^{K}{\left(d_{k}\right)}^{q}\left(c-M(R_{k},R)\right)\\ s.t.\;\left\{ \begin{array}{l} \sum_{k=1}^{K}d_{k}=1\\ d_{k}\geq 0 \end{array}\right. \end{array}$$

in which q is an integer, and q>1.

The similarity $M(R_{k},R)$ is an important component of the model in Formula (1), so we first introduce the similarity measurement of the Vague value.

The Vague value is expressed as [t,1−f], in which t is the truth-membership function denoting the lower bound of the membership degree supporting the evidence, and f is the false-membership function denoting the lower bound of the membership degree opposing the evidence. 0≤t+f≤1. In the following discussion, $x=[t_{x},1-f_{x}]$, $y=[t_{y},1-f_{y}]$. Some similarity measurements have been proposed. The typical methods include *M_C_* proposed by Chen [16], *M_H_* proposed by Hong [17], *M_L_* proposed by Li [18] and *M_Z_* proposed by Zhou [19]. We propose a new similarity measurement by synthesizing the characteristics of existing methods, which covers the distance of the interval’s ends ($\left|t_{x}-t_{y}\right|$ and $\left|f_{x}-f_{y}\right|$), the kernel distance (|t−f|) and the hesitancy degree (π=1−t−f).

The similarity measurement M(x,y) between Vague value x and y is defined as:

(2) $$M_{B}(x,y)=1-{\left(\frac{t_{x}-t_{y}-(f_{x}-f_{y})}{4\sqrt{2}}\right)}^{2}-{\left(\frac{t_{x}-t_{y}+f_{x}-f_{y}}{2\sqrt{2}}\right)}^{2}-{\left(\frac{2-t_{x}-t_{y}-f_{x}-f_{y}}{4\sqrt{2}}\right)}^{2}-{\left(\frac{\left|t_{x}-t_{y}\right|+\left|f_{x}-f_{y}\right|}{4}\right)}^{2}$$

The similarity measurement between *x* and *y* need to meet the Properties (1)–(3) [20]:

Property (1) 0≤M(x,y)≤1;
Property (2) M(x,y)=1 if x=y;
Property (3) M(x,y)=M(y,x).

The measurement in Formula (2) is proven to meet the Properties (1)–(3).

**Proof.**  For Property (1), $∵t_{x},t_{y},f_{x},f_{y}\in [0,1]$,

$\begin{array}{cc} ∴M_{B}(x,y) & =1-\frac{\left|t_{x}-t_{y}-(f_{x}-f_{y})\right|}{16}-\frac{\left|t_{x}-t_{y}+f_{x}-f_{y}\right|}{4}-\frac{2-t_{x}-t_{y}-f_{x}-f_{y}}{16}-\frac{\left|t_{x}-t_{y}\right|+\left|f_{x}-f_{y}\right|}{8}\\ & \leq 1-\frac{0}{16}-\frac{0}{4}-\frac{0}{16}-\frac{0}{8}=1.\;(\text{The equal sign is obtained when}\;t_{x}=t_{y}\;\text{and}\;f_{x}=f_{y}) \end{array}$

$∵\left|t_{x}-t_{y}-(f_{x}-f_{y})\right|\leq 2,\;\left|t_{x}-t_{y}+f_{x}-f_{y}\right|\leq 2,\;2-t_{x}-t_{y}-f_{x}-f_{y}\leq 2,\;\left|t_{x}-t_{y}\right|+\left|f_{x}-f_{y}\right|\leq 2,$

$\begin{array}{cc} ∴M_{B}(x,y) & =1-\frac{\left|t_{x}-t_{y}-(f_{x}-f_{y})\right|}{16}-\frac{\left|t_{x}-t_{y}+f_{x}-f_{y}\right|}{4}-\frac{2-t_{x}-t_{y}-f_{x}-f_{y}}{16}-\frac{\left|t_{x}-t_{y}\right|+\left|f_{x}-f_{y}\right|}{8}\\ & \geq 1-\frac{2}{16}-\frac{2}{4}-\frac{2}{16}-\frac{2}{8}=0.\;(\text{The equal sign is obtained when}\;x=[1,1],\;y=[0,0]\;\text{or}\\ & x=[0,0],\;y=[1,1]). \end{array}$

$∴0\leq M_{B}(x,y)\leq 1$.

For Property (2), set x=y, that is $t_{x}=t_{y}$, $f_{x}=f_{y}$, then

$\begin{array}{cc} M_{B}(x,y) & =1-\frac{\left|t_{x}-t_{y}-(f_{x}-f_{y})\right|}{16}-\frac{\left|t_{x}-t_{y}+f_{x}-f_{y}\right|}{4}-\frac{2-t_{x}-t_{y}-f_{x}-f_{y}}{16}-\frac{\left|t_{x}-t_{y}\right|+\left|f_{x}-f_{y}\right|}{8}\\ & =1-\frac{\left|t_{x}-t_{x}-(f_{x}-f_{x})\right|}{16}-\frac{\left|t_{x}-t_{x}+f_{x}-f_{x}\right|}{4}-\frac{2-t_{x}-t_{x}-f_{x}-f_{x}}{16}-\frac{\left|t_{x}-t_{x}\right|+\left|f_{x}-f_{x}\right|}{8}=1, \end{array}$

∴M(A,B)=1 if A=B.

For Property (3),

$M_{B}(y,x)=1-\frac{\left|t_{y}-t_{x}-(f_{y}-f_{x})\right|}{16}-\frac{\left|t_{y}-t_{x}+f_{y}-f_{x}\right|}{4}-\frac{2-t_{y}-t_{x}-f_{y}-f_{x}}{16}-\frac{\left|t_{y}-t_{x}\right|+\left|f_{y}-f_{x}\right|}{8},$

$M_{B}(x,y)=M_{B}(y,x)$ can be obtained after arranging. ☐

The modified similarity measurement accounts for the hesitancy degrees $\left|\pi_{x}-\pi_{y}\right|$ and $\pi_{x}+\pi_{y}$ in the meantime. $\left|\pi_{x}-\pi_{y}\right|$ is the distance of the hesitancy degree which is smaller when the similarity is greater. $\pi_{x}+\pi_{y}$ means that the overall content of the uncertain information can reduce the similarity when its amount is large. The modified similarity measurement can increase the ability to compare the Vague value with more influence factors, and it can improve the similarity’s precision and differentiation with the power exponent.

Now we consider the solution to the optimization model in Formula (1), and we give the conclusion as follows:

(D,R) is the local minimum solution to Formula (1) when and only when

(3) $$R=\frac{1}{(4+\sqrt{2})\sum_{k=1}^{K}{\left(d_{k}\right)}^{q}}\sum_{k=1}^{K}\left({\left(d_{k}\right)}^{q}(2+\sqrt{2})R_{k}\right)$$

(4) $$d_{k}=\frac{{\left(1/\left(c-M(R_{k},R)\right)\right)}^{1/\left(q-1\right)}}{\sum_{k=1}^{K}{\left(1/\left(c-M(R_{k},R)\right)\right)}^{1/\left(q-1\right)}}$$

**Proof.**  Set *R* fixed and

(5) $$\min g_{q,c}(D)=\sum_{k=1}^{K}{\left(d_{k}\right)}^{q}\left(c-M(R_{k},R)\right),$$

Setting $I(D)=\sum_{k=1}^{K}d_{k}-1$, the solution of Formula (1) is the stationary point of the following Lagrange function in the constraint I(D)=0:

(6) $$L(D,\lambda )=g_{q,c}(D)-\lambda I(D)$$

in which λ is Lagrange multiplier. The stationary point of L(D,λ) meets

(7) $$\frac{\partial L}{\partial \lambda }(D,\lambda )=I(D)=0$$

(8) $$\nabla g_{q,c}(D)=\lambda \nabla I(D)$$

Formula (8) equals

(9) $$\frac{\partial g_{q,c}}{\partial d_{k}}=q{\left(d_{k}\right)}^{\left(q-1\right)}(c-M(R_{k},R))=\lambda \frac{\partial I}{\partial d_{k}}$$

Therefore,

(10) $$d_{k}={\left(\frac{\lambda }{q}\right)}^{1/\left(q-1\right)}{\left(\frac{1}{c-M(R_{k},R)}\right)}^{1/\left(q-1\right)}$$

According to Formula (8),

(11) $$\sum_{k=1}^{K}d_{k}=\sum_{k=1}^{K}{\left(\frac{\lambda }{q}\right)}^{1/\left(q-1\right)}{\left(\frac{1}{c-M(R_{k},R)}\right)}^{1/\left(q-1\right)}=1$$

that is,

(12) (λq)1/(q−1)=1/∑k=1K(1c−M(Rk,R))1/(q−1)

Formula (12) is substituted into Formula (9), then

(13) $$d_{k}=\frac{{\left(1/\left(c-M(R_{k},R)\right)\right)}^{1/\left(q-1\right)}}{\sum_{k=1}^{K}{\left(1/\left(c-M(R_{k},R)\right)\right)}^{1/\left(q-1\right)}}$$

Formula (4) is proven.

Set *D* fixed, $R_{k}=[t_{k},1-f_{k}]$, R=[t,1−f]. According to Formula (1), set

$h_{c}(R)=\sum_{k=1}^{K}{\left(d_{k}\right)}^{q}\left(c-M(R_{k},R)\right)$

(14) $$\begin{array}{l} =\sum_{k=1}^{K}{\left(d_{k}\right)}^{q}(c-1+\frac{1}{n}\sum_{i=1}^{n}({\left(\frac{t_{k}-t-(f_{k}-f)}{4\sqrt{2}}\right)}^{2}+{\left(\frac{t_{k}-t+f_{k}-f}{2\sqrt{2}}\right)}^{2}\\ +{\left(\frac{2-t_{k}-t-f_{k}-f}{4\sqrt{2}}\right)}^{2}+{\left(\frac{\left|t_{k}-t\right|+\left|f_{k}-f\right|}{4}\right)}^{2})) \end{array}$$

*R* must meet $\nabla h_{c}=0$ if we want to obtain the local minimum of $h_{c}$, that is

(15) $$\frac{\partial h_{c}}{\partial R}=\frac{1}{n}\sum_{k=1}^{K}{\left(d_{k}\right)}^{q}\left((2+\sqrt{2})R_{k}-(4+\sqrt{2})R\right)=0$$

Therefore,

(16) $$R=\frac{1}{(4+\sqrt{2})\sum_{k=1}^{K}{\left(d_{k}\right)}^{q}}\sum_{k=1}^{K}\left({\left(d_{k}\right)}^{q}(2+\sqrt{2})R_{k}\right)$$

Formula (3) is proved.

Furthermore, $Q_{q,c}$ can reach the local minimum by improving itself from the direction of *D* or *R*, if (D,R) is not the local minimum of $Q_{q,c}$. Actually, the local minimum is also the global minimum of $Q_{q,c}$ because $Q_{q,c}$ is the convex set and its Hesse matrix is positive definite. Then, we can conclude that Formulas (17) and (18) approximately converge to Formulas (3) and (4) when t→∞.

(17) $$R^{\left(t+1\right)}=\frac{1}{(4+\sqrt{2})\sum_{k=1}^{K}{\left(d_{k}^{t}\right)}^{q}}\sum_{k=1}^{K}\left({\left(d_{k}^{t}\right)}^{q}(2+\sqrt{2})R_{k}\right)$$

(18) $$d_{k}^{\left(t+1\right)}=\frac{{\left(1/(c-M(R_{k},R^{\left(t+1\right)}))\right)}^{1/(q-1)}}{\sum_{k=1}^{K}{\left(1/(c-M(R_{k},R^{\left(t+1\right)}))\right)}^{1/\left(q-1\right)}}$$

The individual’s relative consistent degree can be obtained from the solution of Formula (1). Then, the aggregation coefficient $H_{k}$ can be calculated:

(19) $$H_{k}=\beta \omega_{k}+(1-\beta )\frac{d_{k}}{\sum_{k=1}^{K}d_{k}}\;,0\leq \beta \leq 1$$

in which $\omega_{k}$ is the individual’s weight, and β can adjust the proportion between the individual’s weight and the consistent degree.

The individuals’ opinions can be aggregated with the aggregation coefficient:

(20) $$R=\sum_{k=1}^{K}\left(H_{k}\;\times \;R_{k}\right)$$

☐

### 2.3. Evaluation of Approaches Based on Vague Value

All the alternatives are evaluated from different indexes based on the approach-index matrix to reflect the alternatives’ ability on indexes. The evaluations from some experts are aggregated with the aggregation method of group opinions in this paper.

#### 2.3.1. Approach-Index Matrix

Set *A* is the alternative set $A=\{a_{1},a_{2},\;\cdots ,a_{m}\}$, *C* is the index set $C=\{c_{1},c_{2},\;\cdots ,c_{n}\}$, and *E* is the expert set $E=\{e_{1},e_{2},\;\cdots ,e_{l}\}$. $r_{ij}^{k}$. means the improvement degree of the *i*-th alternative to the *j*-th index, which is evaluated by the *k*-th expert. Each expert’s evaluation opinion can be expressed in a matrix in the form of a Vague value:

(21) $$R^{k}=\left[\begin{array}{ccc} r_{11}^{k} & \ldots & r_{1n}^{k}\\ \vdots & \ddots & \vdots \\ r_{m1}^{k} & \cdots & r_{mn}^{k} \end{array}\right]=\left[\begin{array}{ccc} \left[t_{11}^{k},t_{11}^{k*}\right] & \ldots & \left[t_{1n}^{k},t_{1n}^{k*}\right]\\ \vdots & \ddots & \vdots \\ \left[t_{m1}^{k},t_{m1}^{k*}\right] & \cdots & \left[t_{mn}^{k},t_{mn}^{k*}\right] \end{array}\right]$$

in which $1-f_{ij}=t_{ij}^{*}$, $0\leq t_{ij}+f_{ij}\leq 1$, 1≤i≤m, 1≤j≤n, 1≤k≤l.

The expert’s evaluation opinion can be indicated with a grade variable [21]. The transformation from linguistic variables to Vague values is shown in Table 1.

#### 2.3.2. Evaluation of Approaches Based on Aggregated Opinion

The concrete process of aggregating the experts’ opinions is based on the method in Section 2.2.

(1) For *l* approach-index matrices, the index is fixed as *c_j_*, and *l* experts’ evaluation opinions are transferred to the expert-approach matrix:
  (22) $$EA^{j}={\left[\begin{array}{c} R_{1}\\ \vdots \\ R_{l} \end{array}\right]}_{j}=\left[\begin{array}{ccc} r_{1j}^{1} & \cdots & r_{mj}^{1}\\ \vdots & \ddots & \vdots \\ r_{1j}^{l} & \cdots & r_{mj}^{l} \end{array}\right]$$
  in which each row means the evaluation opinions of *m* alternatives from an expert.
(2) For the matrix in Formula (22), the initial consistent weight is $d_{k}^{\left(0\right)}$, $0<d_{k}^{\left(0\right)}<1$, and $\sum_{k=1}^{K}d_{k}^{\left(0\right)}=1$. The similarity of every two experts is given with Formula (2).
(3) $R^{\left(t+1\right)}$ and $D^{\left(t+1\right)}$ are calculated with Formulas (17) and (18), in which *t* is the iteration step, t=0,1,2, ⋯. The iteration is finished if $||D^{\left(t+1\right)}-D^{\left(t\right)}||\leq \varepsilon$. Otherwise this step is repeated with t=t+1.
(4) Setting $D^{\left(t+1\right)}=\left(d_{1},d_{2},\;\cdots ,d_{l}\right)$, the experts’ aggregation coefficient *H_k_* is calculated with Formula (19).
(5) The experts’ aggregated opinion in the *j*-th index is calculated with Formula (20):
  (23) $$R_{j}=\sum_{k=1}^{l}\left(H_{k}\times R_{k}\right)$$
  in which *R_j_* is the aggregated opinion in the *j*-th index which is a 1×m row vector. Opinions in all indexes are aggregated with the process above. *n* row vectors are combined and transposed to obtain a composite approach-index matrix:
  (24) $$AC=\left[\begin{array}{ccc} r_{11} & \cdots & r_{1n}\\ \vdots & \ddots & \vdots \\ r_{m1} & \cdots & r_{mn} \end{array}\right]$$

### 2.4. Selection of Approaches Based on Sensors’ Monitoring Data

The matrix *AC* indicates the alternatives’ abilities to improve the indexes, which is the general evaluation. In reality, the selected approach should correspond to the real-time status because the monitoring index data are different at different times. The real-time status is reflected by the monitoring data which is also the reference to select the best approach. The final approach is selected by associating the monitoring data with the features of the alternatives. The concrete process is as follows:

(1) To operate with the follow-up index value in real number, the Vague elements in matrix *AC* are transferred to real number with the superiority function:
  (25) $$S(x)=t_{x}-f_{x}+1$$

The matrix *AC* is normalized by rows, $AC\prime =(r_{ij}\prime )$:

(26) $$r_{ij}\prime =\frac{r_{ij}}{\sum_{j=1}^{n}r_{ij}}$$

The row elements in AC′ indicate the alternatives’ abilities in the form of weights. In each row, the higher the element $r_{ij}\prime$, the greater the ability of the alternative to improve the corresponding water index.

(2) The real-time index $x_{j}$ is standardized with q−1 groups of historical data to eliminate the influence of different indexes’ dimensions. The standardization can also reflect the comparison between the real-time and routine indexes.
  (27) $$x_{j}^{*}=\frac{\left|x_{j}-\mu_{j}\right|}{\sigma_{j}},\;j=1,2,\cdots ,n$$
  in which $\mu_{j}$ is the mean value of the sample, $\mu_{j}=\sum_{p=1}^{q}x_{jp}/q$, and $\sigma_{j}$ is the standard deviation of the sample, $\sigma_{j}=\sqrt{\sum_{p=1}^{q}{\left(x_{jp}-\mu_{j}\right)}^{2}/q}$.
(3) The standardized index is denoted as $X=[x_{1}^{*},x_{2}^{*},\;\cdots ,x_{n}^{*}]$. We plan to compare the alternatives’ ability with the real-time index point to point. Then the approach closest to the real-time index trend is selected from the alternatives using the method of the grey relational degree:
  (28) $$l_{ij}=\frac{\underset{1\leq i\leq m}{\min}\left(\underset{1\leq j\leq n}{\min}\left|r_{ij}\prime -x_{j}^{*}\right|\right)+\rho \underset{1\leq i\leq m}{\max}\left(\underset{1\leq j\leq n}{\max}\left|r_{ij}\prime -x_{j}^{*}\right|\right)}{\left|r_{ij}\prime -x_{j}^{*}\right|+\rho \underset{1\leq i\leq m}{\max}\left(\underset{1\leq j\leq n}{\max}\left|r_{ij}\prime -x_{j}^{*}\right|\right)}$$
  in which ρ is the resolution coefficient, and ρ∈(0,1). Set ρ=0.5 to reduce the data distortion caused by the large absolute difference. Then, the grey relational degree is synthesized by row:
  (29) $$l_{i}=\frac{1}{n}\sum_{j=1}^{n}l_{ij}$$
  in which *l_i_* is the relational degree between the *i*-th approach and the real-time index. The higher the relational degree, the more suitable the approach.

The alternatives can be ranked by the relational degree to selected the best approach according to the current condition.

## 3. Example and Results

### 3.1. Remediation Approaches and Indexes of Algal Bloom

The current remediation approaches to algal bloom are divided into three types: physical, chemical and biological methods. The physical methods mainly transfer the pollution source with mechanical equipment or engineering reform, including sewage interception, sediment dredging, mechanical salvage, etc. The chemical methods directly destruct or flocculate the algae with kinds of chemical reagents. The biological methods reduce the amount of algae with biont predation or interspecific competition. The alternative set is constituted of remediation approaches that are common both in the theoretical research and practical application. The alternative set *A* includes mechanical removal (*A*_1_), adsorption (*A*_2_), ultrasonic (*A*_3_), algaecide (*A*_4_), flocculate precipitation (*A*_5_), electrochemistry (*A*_6_), hydrophyte (*A*_7_), microorganism (*A*_8_), and algophagous method (*A*_9_).

The influence factors in the development of algal bloom have been studied widely. Several main factors were chosen to form the index set *C* which includes pH (*C*_1_), total phosphorus (TP) (*C*_2_), total nitrogen (TN) (*C*_3_), chlorophyll a (chl_a) (*C*_4_), dissolved oxygen (DO) (*C*_5_).

Four experts in this field are invited to evaluate the ability of the alternatives to improve the indexes. Then, the initial approach-index matrix *R* is formed (Table 2).

### 3.2. Aggregation of Group Experts’ Opinions

For the approach-index matrixes in Table 2, experts’ opinions are aggregated in the order of indexes. For example, first considering the index *C*_1_ (pH), the expert-approach matrix in Formula (22) is formed:
*EA*^1^=[0.2, 0.3][0.1, 0.15][0.1, 0.15][0.4, 0.6][0.3, 0.45][0.5, 0.5][0.6, 0.8][0.6, 0.8][0.5, 0.5][0.3, 0.45][0.2, 0.3][0.2, 0.3][0.5, 0.5][0.2, 0.3][0.6, 0.8][0.7, 0.85][0.5, 0.5][0.4, 0.6][0.3, 0.45][0.2, 0.3][0.2, 0.3][0.5, 0.5][0.4, 0.6][0.6, 0.8][0.7, 0.85][0.7, 0.85][0.6, 0.8][0.3, 0.45][0, 0][0.3, 0.45][0.5, 0.5][0.5, 0.5][0.4, 0.6][0.5, 0.5][0.5, 0.5][0.4, 0.6]

The opinions in *EA*^1^ are calculated iteratively with the method in Section 2.2 to obtain the experts’ relative consistent degree *D* and aggregation coefficient *H*, as shown in Table 3.

Experts’ opinions in the first index are aggregated following Formula (23) to obtain the comprehensive opinion *R*_1_:

$$R_{1}=[\begin{array}{l} \begin{array}{cccc} \left[0.279,0.419\right] & \left[0.118,0.178\right] & \left[0.210,0.315\right] & \left[0.479,0.520\right] \end{array}\\ \begin{array}{ccc} \left[0.349,0.448\right] & \left[0.518,0.678\right] & \begin{array}{cc} \left[0.618,0.733\right] & \begin{array}{cc} \left[0.558,0.627\right] & \left[0.458,0.617\right] \end{array} \end{array} \end{array} \end{array}]$$

The opinions in the other four indexes are calculated using the steps above to obtain the aggregated opinions *R*_2_, *R*_3_, *R*_4_, and *R*_5_. The five row vectors (*R*_1_ to *R*_5_) are combined and transposed to form the approach-index matrix *AC*, shown in Table 4.

### 3.3. Processing of Real-Time Data and Selection of Remediation Approach

The water quality data are from the Yuyuantan Lake in Beijing. The indexes chl_a, DO, and pH are monitored by the water quality sensor YSI 6600V2. The indexes TP and TN (calculated with ammonia nitrogen) are monitored by the portable tester COD-304. The sensors are set for the same time and frequency. The latest data are standardized with Formula (27). The historical data (49 groups), real-time data (the 50th group) and standardization values are shown in Table A1 in the Appendix Section.

**Figure 2:**
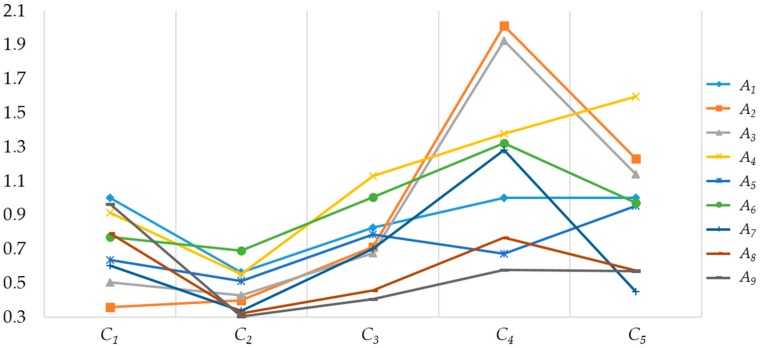
The point relational degree between each alternative and the index data.

**Figure 3:**
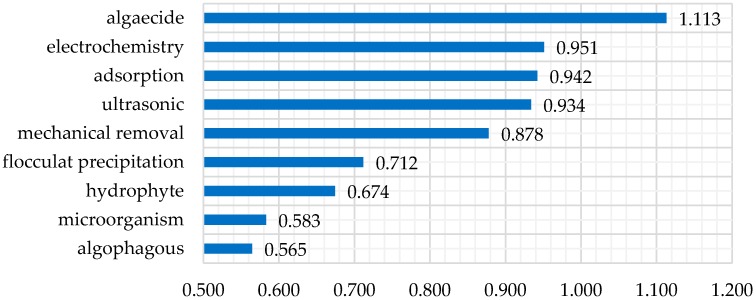
Rank of alternatives. The larger the value, the better the approach.

The elements in approach-index matrix *AC* are transformed into real numbers using the superiority function to form the new matrix *AC’*. The approach-index matrix with real numbers is shown in Table 5. Each row in *AC’* is compared with the index x* using the relational degree. The relational degree between *r’* in *AC’* and x* is calculated with Formulas (28) and (29). The relational degree is the basis for evaluating the alternatives. The result in Figure 2 shows the point relational degree between the alternatives and the index, while Figure 3 shows the total relational degree and the rank result.

From the rank of alternatives, the algaecide method is better than the electrochemistry, adsorption and ultrasonic methods, while the algophagous and microorganism method are the worst. Analyzing the water quality data in Table 5, TP and TN significantly exceed the standard in real time. They can be improved using the algaecide method, so that the method ranks at the front. The indexes exceeding the limits also include chl_a which can be improved by electrochemistry and adsorption method, which therefore rank highly. However, the emphasis of the algophagous and microorganism method is on removing algae macroscopically, which does not match the real-time water quality indexes well. The result shows that the selection model can select the algal bloom remediation approach which best meets the current water quality.

## 4. Discussion and Conclusions

Based on decision-making strategies in water environment management, the fusion of monitoring data and fuzzy evaluation information was explored in this paper. The Vague set and a real number-based group decision-making method were proposed thereafter. Specifically, the similarity measurement of Vague value was modified, and an optimal model was presented to aggregate the group opinions of decision-making experts. Real-time monitoring data from various sensors were associated with the evaluation information to serve the decision-making process and help select the appropriate approach according to the real-time status. The effectiveness of the proposed method was verified in the remediation of algal bloom. 

The characteristics of this method can be summarized as follows:

(1) The novel decision-making method combines the sensors’ monitoring data with the fuzzy evaluation information. It quantifies the qualitative information, and then realizes the fusion of different types of data.
(2) Group opinions of decision-making experts were aggregated in the thought of optimal similarity aggregation. The inconsistency between the aggregated opinion and the original opinions was minimized using the optimal model, making the aggregated opinion more comprehensive.
(3) On the premise of the Vague set, problems of traditional methods applied in the interval number environment were solved by the proposed method. It enhances the expansibility of the classical methods in real numbers while also preserving the advantage of Vague set theory.

The group decision-making method helps to increase the rationality of the remediation of algal bloom. As the basis of the group decision-making process, this paper explores the process of fuzzy information, which is becoming a hot topic in research, such as for the transformation of estimation scales, the comprehension of natural language, and more. Scholars apply granular computing to the process of fuzzy information to solve problems [22,23]. Fuzzy information provides new solutions to group decision-making by combing fuzzy reasoning, artificial neural networks and expert systems with granulating. The method in this paper and other new solutions will help to solve the decision-making method for algal bloom remediation.

## Figures and Tables

**Table 1:** The 11-grade linguistic variables in Vague values.

No.	Grade	Classical Vague Value	No.	Grade	Classical Vague Value
1	absolutely high (AH)	[1, 1]	7	medium low (ML)	[0.4, 0.6]
2	very high (VH)	[0.9, 0.95]	8	fairy low (FL)	[0.3, 0.45]
3	high (H)	[0.8, 0.9]	9	low (L)	[0.2, 0.3]
4	fairly high (FH)	[0.7, 0.85]	10	very low (VL)	[0.1, 0.15]
5	medium high (MH)	[0.6, 0.8]	11	absolutely low (AL)	[0, 0]
6	medium ( M)	[0.5, 0.5]			

**Table 2:** Initial approach-index matrix.

	Expert 1					Expert 2					Expert 3					Expert 4				
	***C*_1_**	***C*_2_**	***C*_3_**	***C*_4_**	***C*_5_**	***C*_1_**	***C*_2_**	***C*_3_**	***C*_4_**	***C*_5_**	***C*_1_**	***C*_2_**	***C*_3_**	***C*_4_**	***C*_5_**	***C*_1_**	***C*_2_**	***C*_3_**	***C*_4_**	***C*_5_**
*A*_1_	L	M	M	VH	H	FL	ML	ML	H	FH	FL	MH	MH	AH	VH	FL	FL	MH	AH	H
*A*_2_	VL	ML	ML	H	VH	L	L	FL	FH	H	L	M	M	VH	AH	AL	L	M	H	VH
*A*_3_	VL	ML	ML	FH	M	L	FL	FL	MH	ML	L	M	M	H	MH	FL	L	FL	FH	M
*A*_4_	ML	FH	FH	FH	FH	M	MH	MH	MH	MH	M	H	H	H	H	M	M	FH	FH	MH
*A*_5_	FL	MH	MH	M	MH	L	M	M	ML	M	ML	FH	FH	MH	FH	M	FH	MH	ML	M
*A*_6_	M	H	FH	FH	H	MH	FH	MH	H	FH	MH	VH	H	H	VH	ML	VH	H	FH	FH
*A*_7_	MH	M	MH	H	FL	FH	ML	M	FH	L	FH	ML	FH	VH	ML	M	ML	FH	FH	L
*A*_8_	MH	ML	ML	MH	ML	M	FL	FL	M	FL	FH	M	M	FH	M	M	FL	M	MH	M
*A*_9_	M	FL	FL	M	ML	ML	L	L	ML	FL	MH	ML	ML	MH	M	ML	ML	ML	ML	ML

**Table 3:** Experts’ relative consistent degree and aggregation coefficient.

	Expert 1	Expert 2	Expert 3	Expert 4
*d*	0.156	0.357	0.130	0.357
*H*	0.203	0.304	0.190	0.304

**Table 4:** Approach-index matrix after aggregation.

	*C*_1_	*C*_2_	*C*_3_	*C*_4_	*C*_5_
*A*_1_	[0.279, 0.419]	[0.447, 0.584]	[0.513, 0.660]	[0.918, 0.959]	[0.787, 0.893]
*A*_2_	[0.118, 0.178]	[0.317, 0.422]	[0.413, 0.511]	[0.788, 0.894]	[0.887, 0.943]
*A*_3_	[0.210, 0.315]	[0.347, 0.467]	[0.369, 0.500]	[0.688, 0.844]	[0.487, 0.585]
*A*_4_	[0.479, 0.520]	[0.647, 0.769]	[0.691, 0.845]	[0.688, 0.844]	[0.662, 0.831]
*A*_5_	[0.349, 0.448]	[0.613, 0.730]	[0.591, 0.720]	[0.458, 0.617]	[0.562, 0.640]
*A*_6_	[0.518, 0.678]	[0.813, 0.906]	[0.713, 0.856]	[0.749, 0.874]	[0.762, 0.881]
*A*_7_	[0.618, 0.733]	[0.426, 0.573]	[0.613, 0.730]	[0.758, 0.879]	[0.262, 0.393]
*A*_8_	[0.558, 0.627]	[0.369, 0.500]	[0.413, 0.511]	[0.588, 0.718]	[0.413, 0.510]
*A*_9_	[0.458, 0.617]	[0.313, 0.469]	[0.313, 0.469]	[0.458, 0.617]	[0.387, 0.535]

**Table 5:** Approach-index matrix in real numbers.

	*C*_1_	*C*_2_	*C*_3_	*C*_4_	*C*_5_
*A*_1_	0.699	1.033	1.174	1.878	1.681
*A*_2_	0.297	0.740	0.924	1.683	1.831
*A*_3_	0.525	0.815	0.870	1.533	1.073
*A*_4_	1.000	1.417	1.537	1.533	1.493
*A*_5_	0.797	1.344	1.312	1.076	1.203
*A*_6_	1.197	1.720	1.570	1.624	1.643
*A*_7_	1.353	1.000	1.344	1.637	0.655
*A*_8_	1.186	0.870	0.924	1.307	0.923
*A*_9_	1.076	0.783	0.783	1.076	0.923

## Appendix A

The monitoring data from sensors is a part of the decision-making basis. The data used in Section 3.3 are shown in Table A1.

**Table A1:** Water quality data from sensors and the standardization value.

No.	pH	TP (mg/L)	TN (mg/L)	Chl_a (ug/L)	DO (mg/L)	No.	pH	TP (mg/L)	TN (mg/L)	Chl_a (ug/L)	DO (mg/L)
1	8.40	0.09	2.26	3.00	8.38	26	7.90	0.01	1.60	32.27	16.01
2	8.70	0.09	2.26	5.90	9.52	27	7.30	0.01	1.60	35.00	15.48
3	8.10	0.09	2.26	8.80	9.69	28	7.09	0.01	1.60	34.35	13.54
4	8.30	0.06	2.26	8.80	12.45	29	7.10	0.02	1.60	37.67	13.13
5	8.70	0.05	2.26	9.90	14.38	30	7.30	0.03	1.45	42.00	13.24
6	8.80	0.05	2.50	13.50	15.32	31	7.20	0.02	1.45	43.56	13.57
7	8.80	0.05	2.50	15.30	16.45	32	7.10	0.02	1.45	45.12	13.78
8	8.70	0.04	2.25	15.78	16.58	33	7.00	0.02	1.45	41.23	13.29
9	8.90	0.03	2.50	20.56	17.23	34	7.00	0.02	1.30	41.12	13.89
10	8.50	0.03	2.25	25.78	17.42	35	7.00	0.01	1.30	40.23	14.56
11	8.40	0.03	2.26	26.78	16.89	36	7.05	0.01	1.30	35.00	14.96
12	8.50	0.03	2.26	27.23	16.93	37	7.10	0.02	1.20	34.78	14.92
13	8.80	0.02	2.26	28.46	16.24	38	7.20	0.02	0.80	31.26	14.87
14	9.30	0.02	2.26	29.34	15.99	39	7.90	0.02	0.80	42.36	15.78
15	9.10	0.02	2.10	30.35	15.78	40	7.50	0.02	0.80	43.67	15.34
16	8.60	0.01	2.10	32.14	15.34	41	7.04	0.02	0.80	45.78	15.24
17	8.70	0.01	2.10	33.45	14.89	42	7.05	0.02	0.80	46.78	15.79
18	8.70	0.02	1.80	35.12	14.32	43	7.10	0.02	0.80	45.12	14.45
19	8.80	0.02	1.80	36.34	14.56	44	7.09	0.02	0.60	45.87	14.38
20	8.90	0.02	1.80	37.45	14.94	45	7.10	0.03	0.60	45.60	14.40
21	8.90	0.02	1.80	36.56	15.53	46	7.09	0.09	0.60	51.60	15.77
22	8.70	0.02	1.80	35.00	15.23	47	7.10	0.09	0.60	46.80	16.94
23	7.90	0.02	1.80	35.45	15.45	48	7.10	0.09	0.45	45.34	17.56
24	7.80	0.02	1.80	34.56	15.67	49	7.09	0.09	0.45	46.70	18.31
25	7.70	0.01	1.60	34.00	15.80	50	7.05	0.09	0.45	53.80	17.89
						*x**	1.08	2.11	1.75	1.65	1.51
